# Click Detect: A Rapid and Sensitive Assay for Shiga Toxin 2 Detection

**DOI:** 10.3390/bios15120813

**Published:** 2025-12-14

**Authors:** Benjamin M. Thomas, Emma L. Webb, Katherine L. Yan, Alexi M. Fernandez, Zhilei Chen

**Affiliations:** 1 Genetics and Genomics Interdisciplinary Program, Texas A&M University, College Station, TX 77843, USA; benjaminthomas@tamu.edu; 2 Department of Biochemistry and Biophysics, Texas A&M University, College Station, TX 77843, USA; emmalwebb@tamu.edu (E.L.W.); alexifern11@tamu.edu (A.M.F.); 3 Department of Microbial Pathogenesis and Immunology, Texas A&M University, Bryan, TX 77807, USA; kyan87@tamu.edu

**Keywords:** click display, diagnostic, DARPin, nanobody, Shiga toxin, environmental

## Abstract

Shiga toxin-producing *Escherichia coli* (STEC) is a major foodborne pathogen, responsible for severe gastrointestinal disease and hemolytic uremic syndrome (HUS). Here, we report Click Detect, a novel diagnostic platform that leverages click display to efficiently produce sensing probes for sandwich-style antigen detection. Click display is an in vitro protein display technology that generates uniform and covalently linked protein–cDNA conjugates in a simple one-pot reaction format within 2 h. The captured sensing probe can be quantified by standard nucleic acid amplification assays. Using click displayed DARPin (D_#20_) as the sensing probe and a high-affinity nanobody (N_G1_) as the capture reagent, Click Detect reliably detected Shiga toxin 2 (Stx2) at 600 fM by quantitative PCR (qPCR) and 6 pM by loop-mediated isothermal amplification (LAMP). The assay maintained comparable sensitivity in matrices containing up to 40% public swimming pool water or lettuce extract, highlighting robustness for real-world surveillance applications. Key advantages of Click Detect include simple, rapid, and cost-effective (~USD 0.04 per assay) sensing probe preparation, as well as a versatile plug-and-play probe format for detecting other targets. We believe that Click Detect has great potential as a novel sensing platform for food/environmental monitoring and point-of-care diagnostics, with potentially broad applicability to other toxins and protein targets.

## 1. Introduction

Shiga toxin-producing *E. coli* (STEC) is a significant zoonotic foodborne pathogen, causing approximately 265,000 STEC infections and 3600 hospitalizations annually in the United States [1]. Infection with STEC typically results from the consumption of contaminated raw or undercooked food (e.g., meats, vegetables, milk, juice), water, or through direct contact with infected animals or humans. STEC represents a considerable public health concern due to its capacity to cause both outbreaks and isolated cases of bloody diarrhea [2]. The economic impact of STEC infection in the US is estimated to be ~USD 280 million per year [3].

Unfortunately, there is currently no effective treatment for STEC. Approximately 4–15% of STEC cases progress to hemolytic uremic syndrome (HUS), a condition that is characterized by acute renal failure, thrombocytopenia, and microangiopathic hemolytic anemia [4,5,6], with a mortality rate of between 5–20% [7,8]. The pathology of STEC stems from secreted toxins—Shiga toxin 1 (Stx1) and Shiga toxin 2 (Stx2)—both of which belong to the AB5 family of protein toxins and comprise an enzymatically active A subunit and a non-toxic pentameric B subunit [9,10]. Upon secretion, the B subunit binds the carbohydrate moiety of the glycosphingolipid Gb3, which is abundant on the extracellular leaflet of cell plasma membranes, and mediates the translocation of the A subunit into the cell cytosol [11,12,13]. The catalytic A subunit is a ribosomal RNA (rRNA) N-glycosidase and removes a single adenine from the rRNA of the host cell, arresting protein translation and leading to cell death [14]. Importantly, Shiga toxins can also cross the epithelial cell barrier and enter the circulatory system, from where they can travel to the kidneys and damage Gb3-expressing glomerular endothelial cells, leading to the onset of HUS in some individuals [4].

Rapid and accurate detection of STEC is critical for food safety monitoring, environmental surveillance, and clinical diagnostics for patient management and HUS risk prediction. However, accurate point-of-care STEC diagnosis remains a significant challenge. The gold standard for diagnosing STEC is through stool culturing (16–24 h at 37 °C) on sorbitol–MacConkey agar (SMAC). Since *E. coli* O157:H7, one of the most notorious STEC serotypes, cannot ferment sorbitol, they form colorless (pale) colonies that can be easily distinguished from the commensal *E. coli* (pink/red). However, culture on SMAC is slow and misses non-O157 STEC strains, which have been reported to also cause STEC illnesses in many places [15]. Multiple nucleic acid amplification tests (NAATs) that detect Stx1 and Stx2 genes have been developed, which provide extremely sensitive and specific results [16,17,18,19]. However, because NAAT detects DNA, not the toxin itself, a positive test result merely indicates the presence of the gene but not whether an active toxin was produced, potentially leading to false positives.

Since the Shiga toxin proteins are the culprit for the disease, direct detection of toxins has the potential to provide more accurate diagnosis and environmental surveillance. A number of methods have been developed for Shiga toxin protein detection, including ELISA [20], LC-MS [21], mouse bioassays [22], cell-based assays [23,24,25], and translation inhibition assays [26]. The sandwich enzyme-linked immunosorbent assay (ELISA) is one of the most widely used detection methods in clinical laboratories and has been reported to detect ~0.025 ng/mL (0.4 pM) of toxin in ~2.5 h. In a conventional sandwich ELISA, the target protein is “sandwiched” between an immobilized capture antibody and a second sensing antibody that recognizes the same target via a different, non-overlapping epitope. After washing, the detection of the sensing antibody via enzymatic/fluorescent labels or additional enzyme/fluorescent label-conjugated antibodies constitutes the target protein sensing.

To improve the target detection sensitivity of ELISA, immuno-PCR (iPCR) assays were developed in which the sensing antibody is conjugated to a strand of DNA for subsequent detection via NAATs (e.g., conventional and real-time PCR, CRISPR-based detection, and isothermal DNA amplification). Although iPCR has been reported to achieve ~4 orders of magnitude better sensitivity than ELISA [27], the practical application of iPCR is hampered by technical challenges associated with DNA-protein conjugation. Throughout the years, multiple strategies have been developed to produce DNA–protein conjugates (Table 1). While covalent conjugation methods (e.g., using amine-reactive or thiol-reactive crosslinkers) are preferred to form stable DNA–protein conjugates, these reactions are most often not site-specific, resulting in a heterogenous population of conjugates with a broad distribution of DNA-to-protein ratio and significant batch-to-batch variation in detection sensitivity. Additionally, extensive purification is required to remove free (unconjugated) DNA, which can be detected in NAATs and produce false signals, as well as unconjugated proteins, which can compete with conjugated protein for the target, adding labor and cost, in addition to often incurring significant conjugate loss.

Our lab recently developed click display, an in vitro protein display technology that yields covalently linked protein–cDNA complexes in a one-pot format [33]. Starting from double-stranded input DNA, the protein display is completed within 2 h, with the cDNA covalently conjugated to the C-terminus of the linked protein via a puromycin molecule. Unlike other in vitro protein display methods that require multiple intermediate purification steps (i.e., mRNA display [34]) or highly specialized in-house reagents (i.e., cDNA display [35]), key advantages of click display include highly efficient and covalent protein–cDNA conjugation, a simple one-pot reaction format, and employing only reagents that are readily available from commercial sources. Click display was originally developed to facilitate the directed evolution of binders from a large library (10^11^–10^12^) to a target protein of interest. However, the facile generation of site-specific protein–cDNA conjugates naturally lends itself to iPCR applications. In this study, we developed Click Detect to explore the use of click display in the sensing of Stx2 protein. We demonstrate the detection of Stx2 via qPCR with a LoD of 600 fM using a purified click display product. Additionally, considering that field operations often lack the luxury of a thermocycler, we also evaluated the click display detection of Stx2 via loop-mediated isothermal amplification (LAMP) [36] and achieved a LoD of 6 pM. Click Detect is compatible with complex matrices, such as pool water and lettuce, that are often sources of STEC contamination. Given the high sensitivity and great simplicity, Click Detect offers a promising first step toward effective detection of STEC for food safety monitoring and environment surveillance.

## 2. Materials and Methods

### 2.1. Protein Expression and Purification

N_G1_ was expressed in SHuffle^®^ T7 cells (New England Biolabs (NEB), Cat# C3029J, Ipswich, MA, USA). The cells were cultured in Luria–Bertani (LB) broth that was supplemented with 50 μg/mL of kanamycin and induced with isopropyl b-d-1-thiogalactopyranoside (IPTG, final 1 mM, Goldbio, Cat# I2481C, St. Louis, MO, USA) when the culture reached OD600 ~0.5. Proteins were purified using immobilized metal affinity chromatography (IMAC) with gravity Ni-NTA beads (PureCube 100 Ni-NTA Agarose, Cube Biotech, Cat# 74705, Monheim am Rhein, Germany) following the standard protocol. The eluted protein was buffer exchanged into 1xPBS (11.9 mM phosphates, 137 mM NaCl, and 2.7 mM KCl, pH 7.4) and concentrated using an Amicon ultrafiltration column (MWCO 10 kDa, Millipore, Cat# UFC9010, Burlington, MA, USA). The purity was analyzed using a 12% SDS-PAGE stain-free gel (Bio-Rad, Cat# 4568043, Hercules, CA, USA), and all were> 90%. The protein concentration was quantified using the Pierce BCA Protein assay (Thermo Scientific, Cat# PI23227, Waltham, MA, USA). Stx2 was similarly expressed and purified as previously described [29].

### 2.2. Preparation of B1-cDNA

Click display of D_#20_ (B1) was carried out exactly as described previously using the PURExpress In Vitro Protein Synthesis Kit (NEB, Cat# E6800L, Ipswich, MA, USA) with 500 ng of PCR amplified D_#20_ DNA as the template for each 12.5 µL transcription/translation reaction. After reverse transcription, the final product was buffer exchanged into 1xPBS (11.9 mM phosphates, 137 mM NaCl, and 2.7 mM KCl, pH 7.4) using a Zeba Spin Desalting Column (40 kDa MWCO, ThermoFisher Scientific, Cat# PIA57759, Waltham, MA, USA). The resulting B1-cDNA was stored as ‘unpurified’ product at −80 °C in aliquots until use. For ‘purified’ click display product, the reverse transcription product was first desalted via ultrafiltration using Amicon (100 kDa MWCO, Millipore, Cat# UFC510024, Burlington, MA, USA), followed by pull-down using Ni-NTA agarose beads (ThermoFisher Scientific, Cat# PI78605, Waltham, MA, USA), washing, and elution using PBS supplemented with 250 mM imidazole.

### 2.3. Preparation of Biotinylated Proteins

IMAC-purified N_G1_ was biotinylated using EZ-Link™ Sulfo-NHS-LC-Biotin (ThermoFisher Scientific Cat# 21335, Waltham, MA, USA) at a 1:4 molar ratio in PBS with a 30 min incubation period at room temperature, followed by overnight incubation at 4 °C. Excess biotin was removed by passing the mixture through 2 Zeba spin desalting columns (7K, MWCO, ThermoFisher, Cat# PI89882, Waltham, MA, USA) sequentially to collect N_G1_-biotin. BSA-biotin was prepared using the same conditions except that Fraction V Bovine Albumin (Thermo Scientific, Cat# J10857-22, Waltham, MA, USA) was incubated with Sulfo-NHS-LC-Biotin at a 1:20 molar ratio.

### 2.4. Preparation of B2-Beads

To form B2-beads, MyOne (ThermoFisher Scientific Cat# 65601, Waltham, MA, USA) streptavidin-coated magnetic bead slurry was first washed thrice with PTBD (1xPBS supplemented with 0.05% tween-20, 0.2% BSA, and 0.3 mg/mL salmon sperm DNA) and then incubated with 100 nM N_G1_-biotin in PTBD at a 1:12 bead slurry to protein ratio at 4 °C with rotation for 10 min. The supernatant was removed, and the beads were further incubated in 100 nM BSA-biotin in PTBD—at the same volume ratio as previously used—at room temperature for 5 min to block any additional unoccupied streptavidin sites on the beads. After removal of the supernatant, the beads were resuspended in PTBD, aliquoted, and used immediately.

### 2.5. Target Detection

For LoD determination, B1-cDNA was diluted 150-fold in PTBD, mixed with serially diluted Stx2 (in PTBD) at a 9:1 volume ratio, and incubated at room temperature for 30 min. Each 10 µL mixture was then mixed with 50 µL of B2-beads (equivalent of 5 µL bead slurry). After another 30 min of incubation at room temperature with rotation, the beads were pulled down using a magnetic stand, washed 4 times with 250 µL of PTBD, resuspended in 10 µL of 1xNEB ThermoPol buffer (NEB, Cat# B9004S, Ipswich, MA, USA), heated to 95 °C for 5 min, and finally, used for NAATs.

For target detection in environmental samples, B1-cDNA was diluted 75-fold in PTBD and mixed with serially diluted Stx2 and 100% pool water or lettuce extract at a 5:1:4 volume ratio. Pool water was collected from local recreation areas by collecting water that was several cm below the surface. Lettuce extract was obtained by collecting the liquid released by compression of lettuce purchased from a local retailer.

### 2.6. NAATs

For LAMP detection, 1 μL of the resuspended B1-cDNA-Stx2-B2-beads mixture was added to 9 µL of the LAMP mixture (0.2 µM of primers F3/B3, 1.6 µM FIP/BIP, 0.4 µL of LF/LB, 0.4 mM dNTP, 3.2 U of Bst Warmstart^®^ 2.0 DNA polymerase (NEB, Cat # M0537S, Ipswich, MA, USA), 0.2X EvaGreen dye (Biotium, Cat# 31019, Fremont, CA, USA), 6 mM MgSO4 (NEB), and 1×isothermal amplification buffer (NEB)). The reaction was carried out at 65 °C in a qPCR instrument (Bio-Rad CFX Duet Real-Time PCR).

For qPCR detection, 1 μL of the resuspended B1-cDNA-Stx2-B2-beads mixture was added to 9 μL of the qPCR mixture (200 nM qF/qR in 1x Forget-Me-Not™ EvaGreen^®^ qPCR Master Mix (Biotium, Cat #31041, Fremont, CA, USA)). The reaction was carried out in the same qPCR instrument using the following program (95 °C for 2 min, followed by 40 cycles of 95 °C for 15 s, 60 °C for 30 s, and 72 °C for 30 s, and final extension at 72 °C for 5 min). The Ct/Mt values were determined using the regression method from CFX Maestro Version 2.3 software.

## 3. Results

### 3.1. Optimization of the Click Detect Assay for Stx2

**Figure 1:**
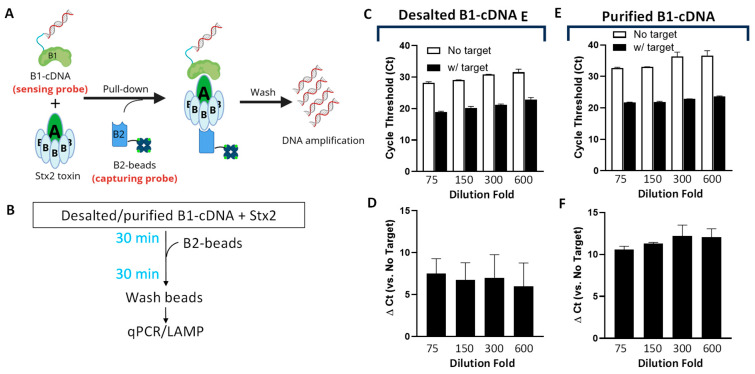
Optimization of the Click Detect assay for Stx2. (**A**) Schematic of Click Detect. Binder 1 (i.e., D_#20_) is linked to its coding cDNA via click display (B1-cDNA) and then incubated with the target Stx2 toxin. Binder 2 (i.e., N_G1_) is biotinylated and immobilized on streptavidin-coated magnetic beads (B2-beads). The presence of Stx2 mediates the pull-down of B1-cDNA by B2-beads and cDNA detection via NAAT assays. (**B**) Diagram of the procedure for Click Detect. (**C**–**F**) qPCR cycle threshold (Ct) of different concentrations of B1-cDNA pulled down by B2-beads from samples incubated in the absence or presence of Stx2 (600 pM). B1-cDNA was either simply desalted (**C**,**D**) or desalted and purified via Ni-NTA beads (**E**,**F**) and serially diluted in PTBD. Ct was determined using regression, and the ∆Ct values are the average from two independent experiments carried out in duplicates. Error bars for Ct represent standard deviation (SD), while the error bars for ∆Ct indicate the standard error of the mean (SEM).

The overall design of Click Detect closely emulates that of iPCR, where two non-competitive Stx2 protein binders are employed (Figure 1A). Binder 1 (B1) is DARPin #20 (D_#20_), a designed ankyrin repeat protein (DARPin) that was previously engineered by us for neutralizing Stx2 toxicity [37]. D_#20_ showed very high binding affinity for the A subunit of Stx2 (EC50 < 1 nM in ELISA). B1 was conjugated to its coding cDNA to form the sensing probe (B1-cDNA). Binder 2 (B2) is a nanobody G1 (N_G1_) and is reported to have high affinity (KD ~23 pM) and specificity toward the B subunit of Stx2 [38]. B2 was recombinantly purified (Figure S1), biotinylated via NHS chemistry, and immobilized on streptavidin-coated magnetic beads to form the capturing probe (B2-beads). B1-cDNA was preincubated with Stx2 for 30 min before the addition of B2-beads (Figure 1B). After washing to remove unbound components, the cDNA pulled down by B2-beads was amplified by NAATs for the detection of the target.

The B1-cDNA conjugate was produced by click display as described previously [33]. For each 12.5 µL of in vitro transcription/translation reaction, 500 ng of input template DNA encoding D_#20_ (Table S2) was used, and the final product after reverse transcription was desalted, resuspended in 150 µL PBS, and stored at −80 °C in single-use aliquots. B2-beads were prepared by immobilizing biotinylated N_G1_ on streptavidin-coated magnetic beads.

Since excess B1-cDNA may bind the streptavidin-coated beads non-specifically and produce false signals, we first determined the quantity of B1-cDNA that yields the optimum signal-to-noise ratio. In the first experiment, B1-cDNA, after reverse transcription, was simply desalted using size-exclusion chromatography with a Zeba column (40 k MWCO), diluted 75–600-fold in PTBD (PBS supplemented with 0.05% (*v*/*v*) Tween 20, 0.2% (*w*/*v*) BSA and 0.3 mg/mL of salmon sperm DNA), and incubated in the presence or absence of Stx2 (final 600 pM) at room temperature for 30 min before the addition of B2-beads. After 30 min, the B2-beads were washed, and the amount of cDNA recovered from the B2-beads was quantified using qPCR. As anticipated, regardless of Stx2, lower dilutions resulted in higher amounts of non-specific binding, manifested as lower-cycle threshold (Ct) values in beads only (no target) samples (Figure 1C). The assays exhibit relatively high variability of the ΔCt values (5–10), calculated from samples incubated in the absence and presence of Stx2 (Figure 1D), likely resulting from input template DNA (estimated to be ~486 kDa).

Since Zeba columns can only remove low-molecular-weight contaminants (e.g., salts and small molecules), the flow-through should retain all the input template DNA, which may contribute to the background NAAT signal. To address this, B1-cDNA, which harbors a 6xHis-tag at the N-terminus (Table S2), was purified via ultrafiltration (100 kDa cut-off) followed by immobilized metal affinity chromatography (IMAC) with Ni-NTA beads. At the same dilution fold, purified B1-cDNA showed a slightly increased Ct for the samples incubated in the presence of Stx2 than desalted B1-cDNA, especially at a lower dilution (Figure 1E). However, the Ct for the samples incubated in the absence of Stx2 increased significantly, resulting in greatly improved Δ Ct between samples containing and lacking Stx2 (Figure 1F). The 150-fold dilution of purified B1-cDNA showed the most consistent ΔCt values between batches and was selected for subsequent assays.

### 3.2. Determination of Stx2 Sensing LoD

**Figure 2:**
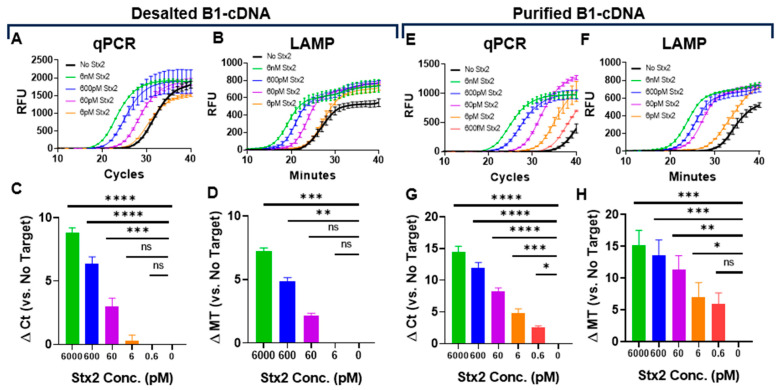
Determination of Stx2 sensing LoD in assay buffers using desalted (**A**–**D**) or purified (**E**–**H**) B1-cDNA. Kinetic curves (**A**,**E**) and calculated Δ Ct (**C**,**G**) of qPCR reactions. Kinetic curves (**B**,**F**) and calculated Δ Mt (**D**,**H**) of LAMP reactions. Ct and minute (Mt) to detection were determined using regression. The kinetic curves are from one representative experiment from three independent experiments. RFU values were averaged across technical replicates. The ∆ Ct or ∆ Mt values are the average from three independent experiments carried out in technical duplicates. The limit of detection was determined using one-way ANOVA (*n* = 3 per group). Error bars indicate SEM, and statistical significance was determined by Fisher’s LSD test versus No Target (*: *p* < 0.05; **: *p* < 0.005, ***: *p* < 0.0005, ****: *p* < 0.0001).

To determine the limit of detection (LoD) of our Stx2 sensing system, we first used desalted B1-cDNA as the sensing probe. B1-cDNA was diluted 150-fold in PTBD and incubated with serially diluted Stx2. Stx2 at 60 pM can be reliably detected by qPCR, although the detection of 6 pM Stx2 was unreliable (Figure 2A,C). Since thermocyclers are often unavailable in point-of-care settings, we also evaluated cDNA quantification by loop-mediated isothermal amplification (LAMP) and confirmed that LAMP can reliably detect Stx2 at 600 pM (Figure 2B,D). The entire qPCR reaction takes approximately 1.5 h to complete, while the LAMP reaction takes less than 40 min (Mt).

Next, we repeated the assays using purified B1-cDNA. Purified B1-cDNA achieved significantly better Stx2 detection sensitivity with a LoD of 0.6 pM for qPCR and 6 pM for LAMP (Figure 2E–H). The improved LoD largely stems from significantly reduced background signals from samples that are incubated with B2-beads in the absence of Stx2 (No Stx2). This is likely due to the absence of free DNA, enabling purified B1-cDNA to have a very low background signal, with Ct ~ 34 in qPCR and Mt ~ 31 in LAMP. In contrast, desalted B1-cDNA exhibits a background signal with Ct ~ 27 and Mt ~ 26. It is somewhat surprising that qPCR achieved slightly better detection sensitivity than LAMP since both have comparable sensitivity (Figure S2). In the current study, the LAMP primer set (Table S3) was designed using the NEB LAMP Primer Design Tool. It is possible that, with better primer design, LAMP detection sensitivity may be improved.

### 3.3. Click Detect of Stx2 in Environmental and Food Matrices

**Figure 3:**
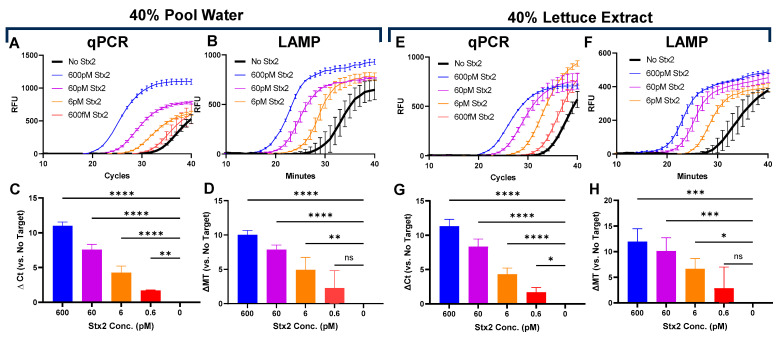
Determination of Stx2 LoD in pool water (**A**–**D**) or lettuce (**E**–**H**). Kinetic curves (**A**,**E**) and calculated ∆ Ct (**C**,**G**) of qPCR reactions. Kinetic curves (**B**,**F**) and calculated ∆ Mt (**D**,**H**) of LAMP reactions. Ct and Mt were determined using regression. Calculated ∆Ct (**C**,**G**) and ∆Mt (**D**,**H**) are the averages from three independent experiments carried out in technical duplicates. The limit of detection was determined using one-way ANOVA (*n* = 3 per group), and statistical significance was determined by Fisher’s LSD test versus No Target (*: *p* < 0.05; **: *p* < 0.005, ***: *p* < 0.0005, ****: *p* < 0.0001).

Challenging matrices often reduce assay sensitivity, limiting their real-world translation. To showcase Click Detect’s potential for environmental monitoring, we determined the Stx2 sensing LoD in an assay buffer that was spiked with swimming pool water and lettuce extract—representative matrices contaminated with chemicals and disinfectants. Purified B1-cDNA was incubated in PTBD spiked with Stx2 and up to 40% *v*/*v* pool water or lettuce extract (Figure 3 and Figure S3). In both cases, similar LoDs were observed, with qPCR achieving a slightly better sensing LoD (0.6 pM) than LAMP (6 pM). Although the Stx2 sensitivity needed for water and food monitoring is unknown, the ability of Click Detect to sense Stx2 in these complex environmental and food matrices underscores the robust nature of Click Detect and its potential for field applications.

## 4. Discussion

STEC is a serious global public health concern, causing sporadic outbreaks [39]. Currently, there is no effective cure for STEC. Although antibiotics can reduce the bacteria load, they can also activate the Stx prophage [40], leading to increased toxin release and worsened patient outcomes. Efficient and frequent food safety monitoring and environment surveillance are, therefore, critical for preventing or limiting the scale of STEC outbreaks, prompting a quicker disease diagnosis and identification of the source(s) of infection.

In this study, we designed and demonstrated a proof-of-concept of Click Detect as a surrogate immuno-PCR/LAMP assay for Stx2 detection. Click Detect takes advantage of click display, which enables facile and rapid preparation of protein–DNA probes in a one-pot format (<2 h) [33]. A key advantage of using click display to prepare the sensing probe is the formation of uniformly linked covalent protein–DNA conjugates, which is difficult to accomplish via conventional amine-/thiol-reactive chemical reactions or streptavidin(tetramer)–biotin reactions. Homogenous protein–DNA probes have the potential to greatly improve assay sensitivity and significantly reduce batch-to-batch assay variation. Similar protein–DNA probes were previously produced using cDNA display technology [41] and demonstrated high assay sensitivity and compatibility with serum samples [41]. Although both cDNA display and click display yield a 1:1 covalent complex of a protein and its coding cDNA via puromycin, the use of click chemistry affords click display a simpler and more efficient linker preparation.

We observed significantly improved detection sensitivity with the Ni-NTA purified protein–DNA sensing probe (i.e., B1-cDNA), mostly through a reduction in background signals (Figure 2). Protein–DNA sensing probes simply desalted via size-exclusion chromatography retain both the input DNA template used for transcription and unconjugated cDNA. Since the primers for qPCR and LAMP cannot distinguish B1-cDNA from the input DNA nor unconjugated cDNA, DNA that non-specifically binds the B2-beads independent of Stx2 can also be amplified, leading to background signals. The 6xHis-tag appended to the N-terminus of B1 enabled efficient removal of the template and unconjugated DNA via one-step immobilized metal affinity chromatography by using Ni-NTA beads and significantly reducing the background signal while improving Stx2 sensing LoD.

In the current format, Click Detect can reliably detect Stx2 at 0.6 pM (~0.05 ng/mL) by qPCR format, and 6 pM (~0.5 ng/mL) by LAMP. This LoD is similar to that of an optimized ELISA (LoD 0.02–0.05 ng/mL) [20]. Since immuno-PCR has been reported to achieve up to five orders of magnitude better LoD than ELISA, the LoD of Click Detect can potentially be significantly improved if paired with alternative binders with better Stx2 binding affinity and specificity. In this study, N_G1_ was used as the capturing reagent and was biotinylated via NHS chemistry. N_G1_ binds the basal lateral site of the B subunit and inhibits Stx2 attachment to host cells [38]. Since the NHS indiscriminately reacts with primary amines on surface-exposed lysine residues, biotinylation of lysine at or near the binding interface on N_G1_ may result in reduced Stx2 binding efficiency. In the future, site-specific biotinylation techniques, such as those employing click chemistry-compatible non-natural amino acids, may be used for N_G1_ biotinylation to improve pull-down efficiency. D_#20_ is a DARPin that was previously engineered for Stx2 neutralization and binds the A subunit of Stx2. We chose to incorporate D_#20_ in the sensing probe, mainly due to its targeting of an epitope distal to the B subunit, which should minimally interfere with the sandwich formation, as well as its lack of a disulfide bond. To date, we have only used the NEB PURExpress in vitro Protein Synthesis Kit for click display of DARPins and, as such, have not attempted to display proteins with disulfide bonds (e.g., single-chain variable fragments (scFvs)). It is possible that alternative in vitro protein synthesis formulations with a disulfide bond enhancer can be used to expand the binder repertoire for the sensing probe.

Contamination of STEC is a persistent issue as there are many reservoirs for STEC and Stx2 in the environment [42]. Leafy greens are among the most widely consumed vegetables and, as such, have been repeatedly associated with STEC outbreaks [43,44]. Sporadic outbreaks stemming from untreated water have also been reported [45]. Click Detect may be suitable for certain environmental tests as it can tolerate up to 40% pool water and lettuce extract with similar LoDs (0.6 pM for qPCR and 6 pM for LAMP) as those in the assay buffer. Another common source for STEC outbreaks in the US is ground beef [46]. Unfortunately, Click Detect failed to robustly detect Stx2 in samples containing >10% beef extract. We suspect that components of beef extract may compromise the integrity of the sensing probe (e.g., DNase), compete for binding to Stx2 (e.g., serum amyloid), or degrade D_#20_/N_G1_ (e.g., protease). However, additional reaction optimization may overcome these hurdles and facilitate Stx2 sensing by Click Detect in these complex samples.

In conclusion, we report the development of Click Detect, a sandwich-style immuno-NAAT assay, for target detection. In Click Detect, the sensing probe, produced by click display, is covalently linked to DNA, uniform in nature, low cost (USD 0.04 per assay), and has fully preserved targeting binding capacity, properties conducive for ultrasensitive immuno-NAAT detection. This contrasts with sensing probes produced via conventional amine-/thiol-reactive crosslinkers that are not site-specific and inherently heterogenous. Although the Stx2 detection LoD (600 fM) achieved by our current Click Detect format is similar to ELISA, we believe the detection sensitivity could potentially be significantly improved when employing alternative Stx2-binders with better affinity and specificity. Additionally, since a plethora of paper-based lateral flow assays have been reported recently for DNA amplification and detection at the point-of-care setting [47,48,49,50], Click Detect may be integrated with these assays to achieve simpler/cheaper target sensing in the future.

## 5. Conclusions

Click Detect offers simple and efficient target sensing and is compatible with complex matrices. We envision that our technology can be easily extended to the sensing of diverse other targets.

## Figures and Tables

**Table 1:** Different strategies for preparing protein–DNA conjugates.

Strategy	Mechanism	Site-Specific	Note
Streptavidin bridge [28]	Biotinylate binder + biotin-DNA; bridge with streptavidin	No	Pros: Fast and high affinity Cons: Multivalency; large complex;
Protein A/G adaptor [29,30]	Binder (IgG Fc) captured by Protein A/G that is itself DNA-labeled	No	Pros: No modification on the antibody binder paratope Cons: Noncovalent
Amine conjugation [31]	Install a reactive moiety on lysine residues; react with functionalized DNA	No	Pros: Simple and robust Cons: Random lysine labeling may impair the paratope
Thiol conjugation [32]	Install reactive moiety on Cys residues; react with functionalized DNA	Yes/No; depends on the number of free Cys	Pros: Small linker, potentially 1:1 conjugation Cons: Requires cysteine engineering
Click Display [33] (This work)	Directly conjugate cDNA to the binder post-translational	Yes	Pros: Fast, site-specific, no modification on binder paratope Cons: Requires binder recombinant expression

## Data Availability

The original contributions presented in this study are included in the article/Supplementary Materials. Further inquiries can be directed to the corresponding author.

## Supplementary Materials

The following supporting information can be downloaded at: https://www.mdpi.com/article/10.3390/bios15120813/s1, Figure S1: Purification of N_G1_; Figure S2: Limit of detection of B1-cDNA template DNA; Figure S3: Environmental buffer screening; Table S1: Amino acid sequence of N_G1_ used in this study; Table S2: DNA and amino acid sequences of B1 (D_#20_); Table S3: Oligos used in this study.

## Abbreviations

The following abbreviations are used in this manuscript:

LAMP	Loop-mediated isothermal amplification
qPCR	Quantitative polymerase chain reaction
STEC	Shiga toxin-producing *Escherichia coli*
HUS	Hemolytic uremic syndrome
LoD	Limit of detection
Stx2	Shiga Toxin 2
B1	Binder 1
B2	Binder 2
NAAT	Nucleic acid amplification tests
iPCR	Immuno-PCR
