# Supplementary material for: Click Detect: A Rapid and Sensitive Assay for Shiga Toxin 2 Detection
Source: Biosensors (Basel). 2025 Dec 14;15(12):813. doi: 10.3390/bios15120813 (PMC12730240; doi:10.3390/bios15120813)
Supplement: Supplementary file 1 [file biosensors-15-00813-s001.zip › biosensors-3948321-supplementary.pdf]

Title: Click Detect: A Rapid and Sensitive Assay for Shiga Toxin 2 Detection

Authors: Benjamin M. Thomas[1], Emma L. Webb[2], Katherine L. Yan[3], Alexi M. Fernandez[2] and Zhilei Chen [1,2,3,*]

Affiliations:

[1] Genetics and Genomics Interdisciplinary Program, Texas A&M University, College Station, TX 77843, USA

[2] Department of Biochemistry and Biophysics, Texas A&M University, College Station, TX 77843, USA

[3] Department of Microbial Pathogenesis and Immunology, Texas A&M University, Bryan, TX 77807, USA

Corresponding author

Zhilei Chen, zchen4@tamu.edu

Supplementary Materials

A

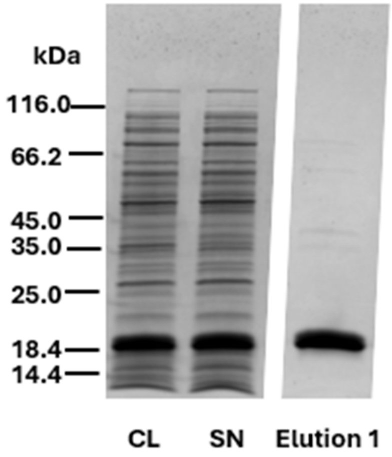

Figure S1. Analysis of purified N_{G1} on SDS-PAGE gel (12%). The gel was stained with Coomassie blue. CL: Crude Lysate, SN: Soluble lysate.

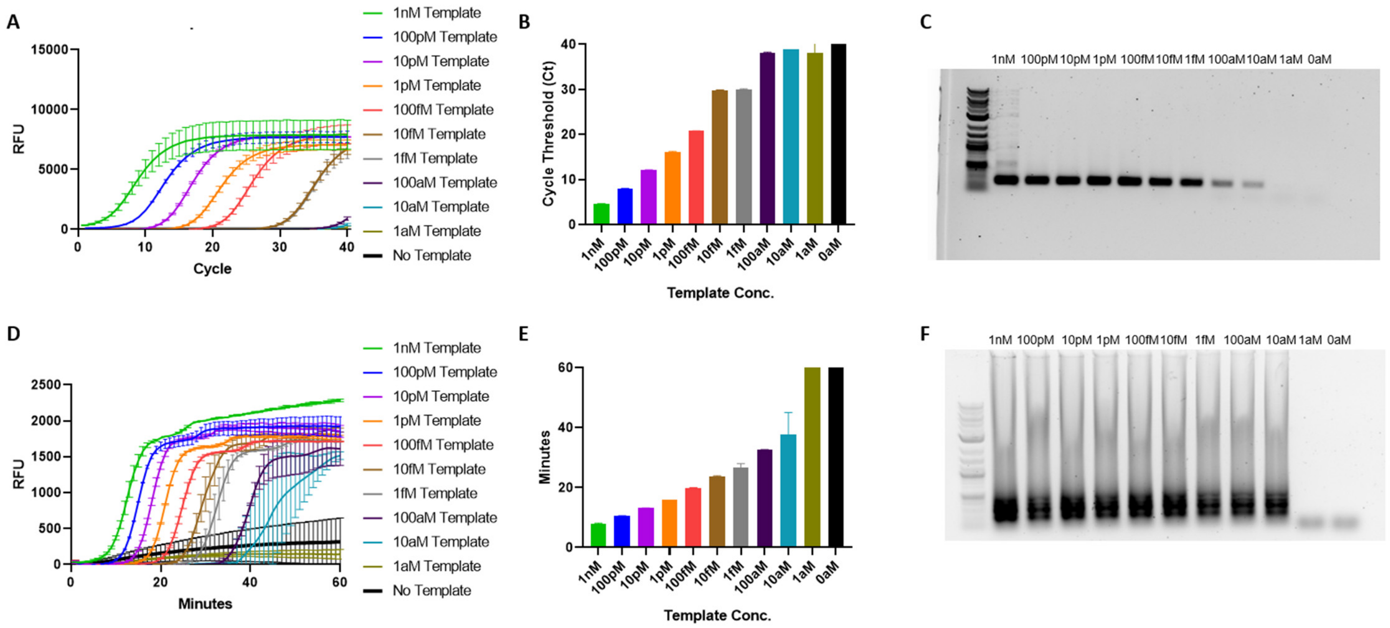

Figure S2. Limit of Detection of B1-cDNA template DNA. Comparison of amplification efficiency of full-length template between qPCR **(A-C)** and LAMP **(D-F).** Representative amplification curves from 2 independent experiments were shown **(A, D)**. Error bars represent the standard deviation of technical replicates. Average cycle threshold **(B)** and minutes to detection **(E)** from two independent experiments. End-point analysis of the amplification products **(C, F)** was performed on 1% agarose gel alongside 1kb Quick-Load ladder (NEB) at 120V for 15 minutes.

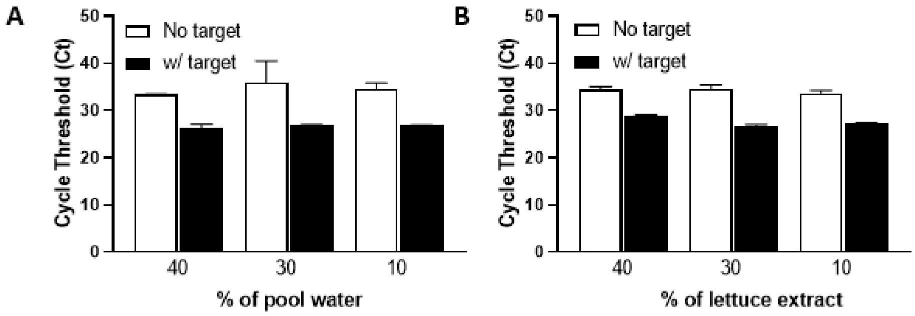

Figure S3. Environmental Buffer Screening. Detection of 600pM Stx2 in 10-40% pool water **(A)** and lettuce extract **(B)**. Efficiency of detection is determined by qPCR.

Table S1: Amino acid sequence of N_{G1} used in this study

Name	Target	Sequences
G1	Stx2a-B subunit	MGSSHHHHHHSSGLVPRGSHMEQKLISEEDLGSQVQLVESGGGLVQPGESL RLSCVASASTFSTSLMGWVRQAPGKGLESVAEVRTTGGTFYAKSVAGRFTIS RDNAKNTLYLQMNSLKAEDTGVYYCTAGAGPIATRYRGQGTQVTVSSAHHSE DPS

Myc-Tag
6xHis Tag

Table S2: DNA and Amino Acid Sequences of B1 ($D_{\#20}$)

DNA Sequence
atacgaaattaatacgactcactataggggagaccacaacggtttccc*tctaga*aataattttgtttaactttaagaaggaggatatatccATGggcagca gccaccatcaccaccatcatcatcacagcagcggcggagaacaaaaactgatcagcgaagaggatctgggatccgatctaggaaaaaagttgttag aagcagctcgcgcaggccaggacgacgaggtgcgtattctgatggcaaatggcgcggacgtgaacgcggcggacccggcaggttggacgccactg cacctggcggcctgctacggccacttagagatcgtgggcgtgctgctgaagaacggcgcggacgttaacgccctggattggggtggttggaccccgct gcacctggctgccgttattggtcaccttgagatcgtcggtgttctgttgaagcatggtgctgacgtgaacgctaccgatctgagcggttggactccgctgcatt tggctgcgtttcgtggtcatttggagatcgttgaagttctgctgaaacacggcgcggacgtcaacgcgcaggataagttcggcaaaaccgcattcgatatt agcattgataatggtaatgaagatttggcggaaatcctccaaagctcctctggtaccggaggtcctactctcctcaaggaaggcaaaaggacgggggg cggcgtggaataactagcataacccctctctaaacggaggggttt

Amino Acid Sequence
MGSSHHHHHHHHSSGGEQKLISEEDLGSDLGKKLLEAARAGQDDEVRILMANG ADVNAADPAGWTPLHLAACYGHLEIVGVLLKNGADVNALDWGGWTPLHLAAVI GHLEIVGVLLKHGADVNATDLSGWTPLHLAAFRGHLEIVEVLLKHGADVNAQDKF GKTAFDISIDNGNEDLAEILQSSSGTGGPTLLKEGKRTGGGVE

T7 promoter
Xba1 recognition sequence
Ribosome binding site
ATG: start codon
ML annealing site
T7 terminator
6xHis Tag
Myc-Tag

Table S3: Oligos used in this study.

Oligo Name	Lab code	Sequence	Function
BIP	3016	GGTCACCTTGAGATCGTCGGTG ACCGCTCAGATCGGTAGC	LAMP primer BIP
B3	3014	CAAATGCAGCGGAGTCCA	LAMP primer B3
FIP	3015	ATAACGGCAGCCAGGTGCAG GGCGTGCTGCTGAAGAAC	LAMP primer FIP
F3	3013	CGGCCACTTAGAGATCGTG	LAMP primer F3
LB	*3018*	GAAGCATGGTGCTGACGTGA	LAMP primer Loop B
LF	*3017*	GGTCCAACCACCCCAATCC	LAMP primer Loop F
qF	*3203*	GTGAACGCTACCGATCTGA	qPCR-F
qR	*3205*	GAGAGTAGGACCTCCGGTA	qPCR-R
